# Patient-Derived Cancer Organoids for Precision Oncology Treatment

**DOI:** 10.3390/jpm11050423

**Published:** 2021-05-17

**Authors:** Mark N. Pernik, Cylaina E. Bird, Jeffrey I. Traylor, Diana D. Shi, Timothy E. Richardson, Samuel K. McBrayer, Kalil G. Abdullah

**Affiliations:** 1 Department of Neurological Surgery, University of Texas Southwestern Medical Center, Dallas, TX 75235, USA; mark.pernik@utsouthwestern.edu (M.N.P.); cylaina.bird@utsouthwestern.edu (C.E.B.); jeffrey.traylor@utsouthwestern.edu (J.I.T.); 2 Department of Radiation Oncology, Harvard Medical School, Brigham and Women’s Hospital and Dana-Farber Cancer Institute, Boston, MA 02215, USA; dshi1@partners.org; 3 Biggs Institute, University of Texas Health Science Center at San Antonio, San Antonio, TX 78229, USA; richardson.te@gmail.com; 4 Children’s Medical Center Research Institute, University of Texas Southwestern Medical Center, Dallas, TX 75235, USA; 5 Simmons Comprehensive Cancer Center, University of Texas Southwestern Medical Center, Dallas, TX 75235, USA; 6 O’Donnell Brain Institute, University of Texas Southwestern Medical Center, Dallas, TX 75235, USA

**Keywords:** organoid, stem cell, cancer, glioblastoma, glioma, oncology, precision medicine

## Abstract

The emergence of three-dimensional human organoids has opened the door for the development of patient-derived cancer organoid (PDO) models, which closely recapitulate parental tumor tissue. The mainstays of preclinical cancer modeling include in vitro cell lines and patient-derived xenografts, but these models lack the cellular heterogeneity seen in human tumors. Moreover, xenograft establishment is resource and time intensive, rendering these models difficult to use to inform clinical trials and decisions. PDOs, however, can be created efficiently and retain tumor-specific properties such as cellular heterogeneity, cell–cell and cell–stroma interactions, the tumor microenvironment, and therapeutic responsiveness. PDO models and drug-screening protocols have been described for several solid tumors and, more recently, for gliomas. Since PDOs can be developed in clinically relevant time frames and share many characteristics of parent tumors, they may enhance the ability to provide precision oncologic care for patients. This review explores the current literature on cancer organoids, highlighting the history of PDO development, organoid models of glioma, and potential clinical applications of PDOs.

## 1. Introduction

Precision oncology seeks to identify genetic, epigenetic, and transcriptomic inter- and intratumoral changes and match cancer therapy based on an individual’s distinctive cancer biology [1,2]. This translational paradigm is dependent on the coordination between clinicians and researchers, wherein both parties use their expertise to evaluate potential drug therapies using patient-specific cancer modeling systems. Traditionally, preclinical models in oncology have relied on two-dimensional cell culture and murine models, which have been a mainstay in attempts to advance basic science discoveries to the clinic through clinical trials [3,4]. However, many of the drugs investigated in traditional preclinical models have yielded suboptimal results in clinical trials, culminating in an FDA approval rate as low as 3% for new oncologic therapies [5].

The failure of new cancer therapies may be due, in part, to the intrinsic limitations of two-dimensional cancer cultures and current animal models. Two-dimensional cancer culture techniques are limited by oligoclonal cell populations, genetic homogeneity, and lack of cell–cell or cell–stroma interactions [3,4,6,7]. Two-dimensional cultures also lose tumor-specific heterogeneity through genetic drift that occurs during long-term culture [8]. Furthermore, certain cancer-associated mutations are under-represented in tumor cell lines, necessitating suboptimal efforts to introduce such mutations ectopically into foreign cellular contexts to enable studies of their function [9,10]. In certain cancers that are highly complex, infiltrating, and heterogeneous (such as glioblastoma and pancreatic cancer), these two-dimensional cell lines lack critical inter- and intratumoral complexity and are unable to reflect the epigenetic complexity that is a signature challenge in developing novel therapeutics [3,4,11,12]. Furthermore, mounting evidence suggests close interdependence between tumors and their microenvironments, including surrounding vasculature, immune cells, and nerves, that are not captured in traditional two-dimensional models [13,14]. Gliomas, which range from grade I to grade IV by means of increasing aggressiveness, are infiltrating tumors that invade the brain parenchyma [15]. Currently, few if any cell lines exist that reflect grade II or grade III gliomas, which invariably progress to higher-grade tumors and are linked to a low median survival [15,16]. This effectively precludes in vitro study of cancer progression in this oncologic subtype. Even in vitro models of higher grades of glioma, such as glioblastoma (WHO grade IV), have significant limitations [4]. The concept of directly deriving cell lines from patient tumors has increased the ability to demonstrate genetic diversity in early preclinical studies. However, these remain challenging cell lines to create, with overall yields of 5–10% when attempting to establish patient-derived high-grade glioma cell lines. Isocitrate dehydrogenase (IDH) mutant oncogenes, which are found in the progression of lower-grade gliomas to higher-grade gliomas, are almost completely absent from patient-derived lines, thus prompting efforts to exogenously express these oncogenes in IDH wild-type tumor lines. While this approach represents an important advance in our ability to study these oncogenes, it also has shortcomings, given that many tumor cell phenotypes are shaped by complex interactions between genomic alterations and epigenetic states that may not be fully recapitulated in engineered cell culture models [17,18,19,20]. This particular concern notwithstanding, 2D cell lines offer clear benefits for cancer biology research. These include their high-throughput capacity, thus enabling systematic investigations of promising therapeutics and large-scale drug target identification efforts. Furthermore, animal models of cancer, particularly murine models, offer a closer replication of clinical microenvironments but are limited by the need for immunodeficient mice, time and resource intensity, oligoclonality, and low-throughput capabilities [3,7,21,22]. Together, the limitations of these models suggest that the development of alternative, complementary models may improve precision oncology care.

The recent development of organoids, defined as in vitro three-dimensional models of human tissues, has allowed for more accurate recapitulation of parent organs [6,7,8,9,10,11,12,13,14,15,16,17]. The available literature describing patient-derived cancer organoids suggests that organoid cancer models consistently reflect in vivo tumor genotypes, phenotypes, intertumor heterogeneity, and cell–cell and cell–stroma interactions and are less resource and time intensive compared to existing mouse models [23,24,25,26,27,28,29,30,31,32]. Organoid cancer models may mitigate challenges presented by existing cancer models and augment the implementation of precision oncology by providing personalized platforms to study each patient’s tumor. Herein, we review the history of organoid model development and the use organoids for cancer modeling, with an emphasis on glioma models. We also discuss the clinical implications of cancer organoid models for precision oncology applications.

## 2. History of Organoid Development

Historically, the term “organoid” referred to any in vitro culture whose characteristics resembled a primary organ. However, within the past 15 years, an organoid has been redefined as a self-organizing three-dimensional structure made from murine or patient-derived stem cells or primary tissue [32,33,34,35]. Advances in stem cell science have facilitated the development of organoid cultures, particularly through the isolation of embryonic and pluripotent stem cells, which provided the tinder for the creation of early original organoid models [36,37,38,39]. Sato et al. (2009) were the first to describe organoids using intestinal stem cells for the induction and perpetuation of a human intestinal organoid [36]. By using Lgr5^+^ intestinal stem cells embedded in an extracellular matrix substitute (Matrigel) on media enriched with r-spondin 1, Noggin, and epidermal growth factor, the group was able to create crypt–villus organoid structures that retained the cellular organization and distribution of newly isolated small-intestinal crypts for over 8 months [36]. Since 2009, organoid models have been used to study the development of several tissues, including the brain, retina, lung, thyroid, blood vessels, small intestine, liver, mesodermal kidney, taste buds, pancreas, and ovaries [32,37,39,40,41,42,43,44]. Notably, Lancaster et al. (2014) were the first to describe cerebral organoid models derived from human pluripotent stem cells that could produce a functional cortex, retina, and choroid plexus with discrete brain regions in a 1–2-month time frame [37,45]. Following this, fused dorsal and ventral cerebral organoids demonstrated directional migration and connectivity of GABAergic neurons [44]. Together, the cerebral organoids created by these groups not only offered distinct advantages over standard neuronal culture models but also showed that creation of non-epithelial organoid models was possible and could recapitulate in vivo tissue architecture.

Several organoid models have now been described with protocols that use induced pluripotent, embryonic, or adult stem cells [26,30,36,37,45,46,47,48,49,50,51,52]. The organoid creation protocols for induced pluripotent stem cells and embryonic stem cells are similar, wherein isolated stem cells are exposed to tissue-specific growth factors to create desired embryoid body-like aggregates [34]. The aggregates are then embedded in a scaffold that acts as the extracellular matrix, most commonly Matrigel or collagen, as this allows the stem cell aggregates to develop the three-dimensional architecture that is critical for the development of tissue polarity from cell–cell and cell–stroma interactions [34,53]. The embedded embryoid bodies are then cultured in media containing various tissue-specific growth factors to produce mature organoids [34]. Alternatively, adult stem cells can also be used to create organoid models, but unlike induced pluripotent and embryonic stem cells, adult stem cells do not require genetic transduction [32,38,54,55]. Therefore, organoids created from adult stem cells can be immediately embedded in an extracellular matrix substitute and cultured in media enhanced with growth factors [32]. The use of stem cells to create organoids, however, is limiting, as many require genetic engineering to produce models of pathologic tissue, which may not recapitulate in vivo disease, prompting groups to create tissue-derived organoid models of disease [56,57].

**Figure 1:**
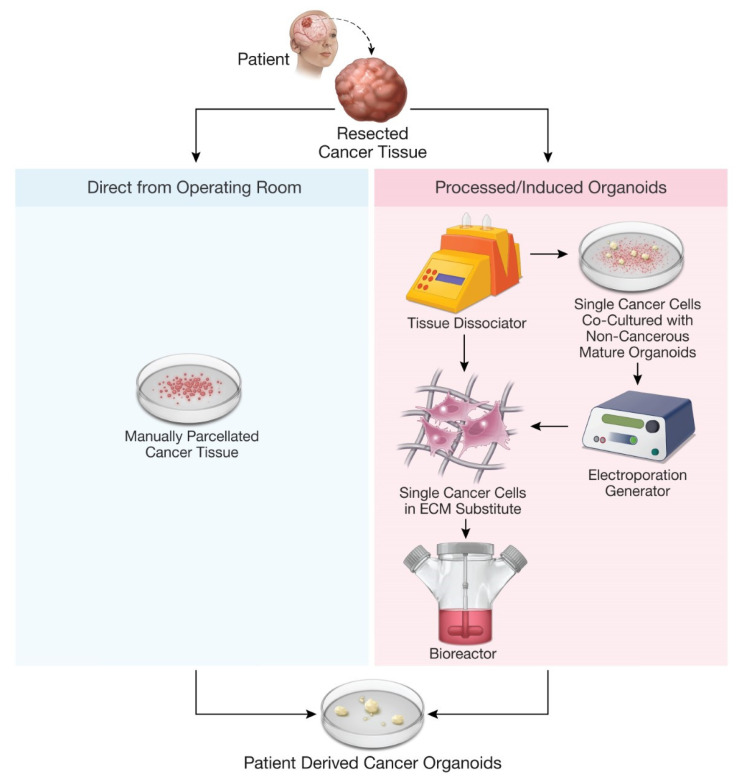
Methods for production of patient-derived organoids.

Organoids have now been developed to model pathologies, including genetic diseases, infectious diseases, parasitic infections, and cancer [8,58]. A primary focus of organoid disease models has been in modeling of cancer, which has resulted in several cancer organoid models derived from epithelial tumors—prostate, liver, pancreas, ovary, bladder, lung, gastrointestinal, and breast, among others (Table 1) [23,24,25,26,27,28,29,30,31,59]. Some of the initial cancer organoid models used genetic modification of stem cells to produce cancer-like organoids, but recent work has focused on developing patient-derived cancer organoid (PDO) models from fresh cancer tissue [26,47,49,56,57,60]. The production of PDOs involves direct coordination between physicians performing tumor biopsy and laboratory personnel in order to create and propagate patient-specific cancer organoids, which may better represent the parent tumor compared to stem-cell-derived cancer organoids [23,24,25,26,27,28,29,30,31,59,61,62]. Biopsied tumor samples used for organoid production are optimally collected from specimens that are highly cellular, not cauterized, necrotic, or marginal samples with surrounding normal tissue as these factors are critical to organoid production success rates and clinical relevance [61,62]. One method to produce PDOs from patient tissue samples entails enzymatic digestion to isolate single cells, especially cancer stem cells, which are then propagated like adult stem cells [35]. While the use of cancer stem cells is one way to create PDOs, this method has been criticized for limiting the clonal heterogeneity of resultant PDOs; hence, most PDO protocols now describe the use of fresh tissue samples that are parcellated but not enzymatically digested to single cells [24,26,30,31,32]. After being parcellated, the tissue samples are cultured in specific media with growth supplements, often embedded in Matrigel, and incubated for weeks to months to produce mature PDOs (Figure 1) [24,26,30,31,32]. For example, pancreatic ductal adenocarcinoma, which remains treatment refractory despite advancements in multimodality therapy, has been modeled with PDOs to test potential treatments [24]. Breast cancer has also been modeled with PDOs, with a landmark study by Sachs et al. (2018) establishing culture conditions necessary for mammary epithelial organoid creation and propagation [28].

Organoids derived from patient tissue appear to have diverse cellular backgrounds that recapitulate parent tumors. PDOs may also maintain the cellular heterogeneity found in human parental tumors [28,29,52,62,63,64,65,66,67,68]. Weeber et al. (2015) demonstrated that 90% of the somatic mutations found in metastatic colorectal cancer biopsy tissue correlated to the corresponding colorectal PDOs [52]. Concordance of patient tumor tissue and PDO heterogeneity has also been demonstrated by several other groups, who found that inter- and intratumoral heterogeneity was maintained in their PDOs based on transcriptomic analysis and exome sequencing [28,29,52,62,65,67,68,69]. The use of immunohistochemical, genomic, and transcriptomic analyses has allowed for characterization of the constituent cells in different PDO models [25,26,27]. Rosenbluth et al. (2020) used mass cytometry to demonstrate that breast cancer organoids maintain important epithelial subpopulations, including basal and luminal progenitor cells, capable of differentiating into all mammary cell lineages in long-term culture [67]. Together, these results indicate that PDOs may capture parent tumor cellular heterogeneity and allow for investigation of unique cell–cell and cell–stroma interactions.

The ability to implement advances in cellular and molecular techniques has facilitated progress in organoid creation. For example, Takebe et al. (2013) created functional, vascularized human liver buds through co-culturing human induced pluripotent stem cells differentiated into hepatic endodermal cells with human umbilical vein endothelial cells and human mesenchymal stem cells [70]. The resultant liver buds produced physiologic levels of albumin 10 days after ectopic transplantation in mice, demonstrating the ability of in vitro functional organ bud development [70]. CRISPR/Cas9 has also been employed by several groups to enhance PDO creation [49,50,68]. Drost et al. (2015) used this method to introduce APC, KRAS, P53, and SMAD4 mutations into mature human small-intestinal stem-cell-derived organoids [49]. The introduction of these mutations allowed human gut stem-cell-derived organoids to grow into colorectal carcinoma organoids with invasive features [49]. Assembloids, combinations of organoids representing different cell populations or regions in an organ, are also used to study the interaction between cellular populations [71,72]. Assembloids can be leveraged, as done by Kim et al. (2020), who created assembloids with patient-derived bladder tumor tissue, cancer-associated fibroblasts, immune and endothelial cells, and muscle to evaluate the epigenetic changes caused by the association of cancer cells with the surrounding stroma and their influence on tumor plasticity [72].

## 3. Current Models of Patient-Derived Cancer Glioma Organoids

Several glioma organoid models have been described in the literature (Table 2). The methods used to create glioma organoids include isolation and culture of patient-derived stem cells, CRISPR/Cas9-mediated mutagenesis of non-cancer cerebral organoids, co-culture of GBM spheroids with normal cerebral organoids, and direct culture of patient-derived glioblastoma samples [56,57,62,82,83]. Hubert and colleagues (2016) first described a GBM organoid model produced from fresh tumor stem cells that recapitulated hallmark features such as hypoxia gradients, selective radiosensitivity, and invasiveness when orthotopically xenografted [57]. The glioblastoma-like organoids created from stem cells with or without directional mutagenesis were also capable of recapitulating some aspects of gene expression and phenotypic behavior of invasive brain tumors [56,68,83,84]. Further, co-culture techniques of glioma stem cells (GSCs) and spheroids with normal cerebral organoids offer significant advantages over xenograft models [82,83,85]. Single-cell RNA sequencing of co-culture of patient-derived GSCs with cerebral organoids demonstrated more accurate representation of the parent tumor compared to glioma spheres, GSC-derived organoids, and xenograft models, indicating maintenance of the tumor microenvironment [85]. However, these stem-cell-based models are limited by the need for exogenous growth factors (which may lead to population drift), reliance on mutagenesis to produce tumor-like phenotypes, use of Matrigel rather than the native extracellular matrix, and relatively slow growth rates [56,57,68,83,84,85].

Jacob et al. (2020) recently described successful GBM organoid production and biobanking from minimally processed fresh GBM samples [61,62]. Their model represents a significant advance from prior models due to several methodologic advantages and biological similarities to human tumors [62]. These GBM organoids were created with fresh tissue samples taken from the operating room in a medium formulated for neural tissue, clipped to appropriate size, and washed with minimal processing [61]. The pieces were subsequently cultured in basal media with added supportive supplements, insulin, and antibiotics and, notably, without an extracellular matrix substitute or exogenous growth factors [61,62]. These organoids formed small, round spheres by 1 week and cell-dense organoids within 2–4 weeks [61,62]. The authors demonstrated that these GBM organoids recapitulated many characteristics intrinsic to human tumors [62]. Their GBM organoids displayed characteristic histologic and immunohistochemical hallmarks of GBM, such as hypoxia gradients, nuclear atypia, and molecular heterogeneity [62,86]. The presence of hypoxia gradients is a hallmark of GBM, as hypoxia-induced signaling through notch and calcineurin pathways is known to contribute to proliferation of GSCs and correlates with patient survival [87,88,89]. RNA and exome sequencing of these organoids demonstrated similarities in gene expression, allele frequency of somatic variants, and similar copy number ratios between the parent tumor and resultant organoids [62]. Intratumor heterogeneity was also preserved in subregion organoid samples, where there was differential expression of gain-of-function epidermal growth factor receptor vIII (EGFRvIII) mutations within single organoids [62,84,90]. Furthermore, these GBM organoids retained the spatial heterogeneity and clustering of neoplastic and immune cells (T cells and macrophages) of their parental tumors, a unique feature that previous models lacked [44,62,68].

Importantly, these GBM organoids exhibited comparable tumor biology to their parental tumors, with similar ex vivo responses to both tumor-specific (tyrosine kinase inhibition) and non-specific (radiation and temozolomide) treatments as compared to their parent tumors [62]. However, the MGMT methylation status did not consistently predict the response to radiation and temozolomide in GBM organoids described by Jacob et al., as is seen in the clinic [62,91]. Lastly, the GBM organoids engrafted into immunodeficient mice within 1–3 months and recapitulated the invasive morphology of human GBM tumors [62]. Moreover, organoid-derived xenografts displayed ipsilateral and contralateral hemispheric invasion, parent-tumor-dependent satellite phenotypes, and neoangiogenesis by host endothelial cells [62]. Recent work by Golebiewska and colleagues (2020) also demonstrated that xenografted glioma organoids maintain numerous genetic glioma subtypes [63]. Together, these results highlight the similarities of histologic and phenotypic properties of GBM organoids to respective parent GBMs, a significant advance from prior glioma organoid models [56,61,62,63,68].

Despite the rapid progress and interval advances in GBM organoid models, numerous limitations and questions remain. First, low success rates in IDH1-mutant (66.7%) and recurrent GBM (75%) indicate additional methodological optimization is needed [61,62]. Further, no data in published research currently exist on the ability to create PDOs from low-grade gliomas, which uniformly progress to higher-grade gliomas (WHO grade III and IV), or non-glioma brain tumors [15,16]. While the immune microenvironment seems to persist in current GBM organoid models, the extent to which it is represented is still in question as we continue to understand GBM–immune interactions [62,92]. The immune microenvironment has historically been difficult to demonstrate in vitro, even in organoid models where most immune cells have not been captured except when artificially replaced [93]. It is also unclear whether nerve–tumor interactions are retained in organoid models, which are a known driver of high-grade glioma proliferation [94]. Data are also underpowered in some experiments, limiting the ability to make definitive conclusions on the success of xenografts for different tumor subtypes and clinical correlation of therapeutic responses [62,63]. Further, glioblastoma is also marked by migration along blood vessels, which has not been demonstrated to date in current xenograft data [95]. Despite the need to address these uncertainties, the existing evidence suggests that patient-derived glioma organoids may offer certain advantages over standard patient-derived cancer models.

## 4. Clinical Applications

The goal of patient-derived cancer cultures is to provide a high-throughput and efficient method to accurately study cancer biology and treatment. Clinically, patient-derived cultures have the potential to help physicians create personalized cancer treatment plans and inform upcoming clinical trials for novel therapies. However, existing in vitro culture models have had limited clinical utility [3,4]. There is mounting evidence indicating that PDOs offer distinct advantages over traditional in vitro methods that can be applied to clinical practice. PDOs can be generated rapidly and faithfully recapitulate characteristics of their parental tumor. These include the ability of PDOs to display similar biomarkers as parent tumors, similarity in treatment responses to human tumors, and ability to be split and propagated for high-throughput drug screening. As such, they may offer the ability to pre-screen multiple therapeutic options in a model system specific to each patient, allowing for individualized treatment selection based on the results of personalized preclinical testing. This is especially valuable for heterogeneous and poor-prognosis tumors such as GBM and pancreatic cancer, where there may be numerous clinical trial options to choose from and few, if any, predictive biomarkers that inform selection among these options.

In the past few decades, there has been a surge in the identification of key biomarkers and mutations that influence cancer progression and are targets for novel treatments. Organoids may offer the ability to recapitulate the parent tumor expression of relevant biomarkers and, in turn, serve as effective surrogates to predict responses to treatment in patients [96]. Numerous studies have reported PDO drug-screening protocols, with preliminary results indicating that organoid drug responsiveness may mimic in vivo responses [28,31,59,62,63,66,79,97]. Sachs and colleagues recently showed that xenografted breast cancer organoids mimic HER2 receptor status and tamoxifen responsiveness to parent tumors [28]. In GBM organoids, Jacobs and colleagues found that drug responsiveness to gefitinib for tumors with EGFR alterations, trametinib for NF-1-mutated tumors, everolimus for PI3K-mutated tumors, and EGFRvIII-targeted CAR-T cells could be generally predicted based on the presence of these mutations in the parent tumor [62]. Importantly, PDO versus clinical outcome studies have demonstrated that even with therapy-dependent biomarkers, drug resistance is recapitulated in subsets of clinically non-responding tumors [28,31,62,63,66,98,99]. This indicates that mutational analysis may not accurately predict drug response [28,31,62,63,66,98,99]. In addition to these examples, drug-screening PDO protocols have been described in pancreatic, gastrointestinal, lung, prostate, ovarian, and bladder cancers [28,29,31,59,62,66,79,97,98,99,100,101]. Reasonably, using PDOs to test treatment efficacy could predict human tumor responsiveness or inform additional research to delineate underpinnings of drug sensitivity. Drug screening in patient-derived organoid models offers advantages in both their similarity to in vivo tumor biology and the increased efficiency of organoid production methodology. Many groups have also described successful biobanking of cancer organoids that can be reanimated from frozen stocks, thus allowing for the development of PDO-matched clinical databases [27,28,29,30,59,61,62].

The increase in efficiency to create PDO models compared to traditional patient-derived cancer cultures makes them well suited for clinical implementation. While there is some variance in production efficiency between tumor types and the cellularity of samples, the establishment of initial organoids tends to take less than 4 weeks, with success ranging from 60% to >90% [30,31,61,62,79,97,100]. Centenera et al. (2018) notably described an efficient patient-derived explant model in which fresh prostate and breast cancer samples could be cultured on gelatin scaffolds within 6 days with high creation success rates [101]. Additionally important for the establishment of numerous organoids from a single patient-derived tumor sample is the splitting and propagation of organoids to facilitate drug-screening protocols, which typically takes between 0 and 7 days for re-establishment, depending on tissue type [97]. The longer time frames needed for the production of previous in vitro models and their lack of representation to parent tumors have rendered these models less clinically useful to guide treatment [3,4] The shortened time frame for PDO production may allow clinicians to initiate drug-screening protocols directly after initial tumor biopsy or resection in order to optimize adjuvant therapy. While the time to complete drug screening will vary based on cancer type, the number of therapies tested, and success rates of organoid establishment, most drug-screening protocols can be completed within 4–6 weeks [97]. Together, organoids and ex vivo models may be especially helpful to guide treatment choices in the setting of pancreatic cancer and GBM, where the median overall survival is short, even with maximal resection and adjuvant therapy [102,103,104]. The low median survival time in aggressive or late-stage cancers creates a narrow window in which physicians can make meaningful changes to treatment modalities. The 1–4-week time frame needed for the creation and propagation of GBM organoids provides an ideal time frame for drug screening to be performed while patients are recovering from primary tumor resection and receiving the universal protocol of temozolomide and concurrent radiation [61,62,102]. Tiriac and colleagues describe a similar drug-screening protocol for pancreatic cancer, which can be completed in as little as 6 weeks, albeit with lower PDO creation success rates (<80%) [66]. High PDO creation success rates will be critical to implementation of screening to ensure that numerous organoids can be propagated from the same tumor efficiently.

Prior to the production of organoids, cancer xenograft models suffered from time intensiveness, low success rates, and lack of representation of polyclonality and phenotypic characteristics [3,4,105]. Xenografts of GBM organoids into mice have shown quick engraftment in 1–3 months with recapitulation of parent tumor characteristics [62,63]. Recently, Golebiewska and colleagues demonstrated that organoid-based glioma xenografts respond predictably to temozolomide, EGFR and CDK4/6 inhibitors, and dianhydrogalactitol, an alkylating agent [62,63]. However, xenograft success rates were lower at 86% for GBM and 25% for grade III gliomas [63]. Conceivably, the production of murine models for gliomas wherein tumor genotypic and phenotypic properties are maintained will allow for quicker testing of preclinical in vivo models. This could speed up clinical trial development and aid in enrollment in ongoing clinical trials. Since PDO xenografts retain important tumor characteristics, drug responses in these models may better represent human tumor responses compared to prior culture techniques [62,63]. However, due to the time to propagate an organoid from a fresh tumor sample (2–4 weeks) and engraft it into a murine model (1–3 months), organoid xenograft models may be challenging to incorporate in bench-to-bedside individualized precision care [22,59,61,62,63].

In the future, organoids may also help inform the development of and enrollment in clinical trials. A major limitation in the progress of human oncologic trials has been the use of preclinical models that may not represent in vivo tumor characteristics [3,4,66]. The biological discordance between heterogeneous human tumors and classical preclinical xenograft models used to test experimental therapeutics may conceivably contribute to therapy failure in clinical trials [3,11,106,107]. The high-throughput capabilities allowed by PDOs could be leveraged to select robustly effective therapies that will succeed in clinical trials via testing numerous biobanked PDOs as opposed to fewer xenografts. Further, PDOs could act as a “pre-screen” to clinical trial enrollment, wherein a patient’s PDOs are screened for a response to the novel therapy prior to enrollment. Together, these strategies may lead to improved preclinical drug screening and clinical trial patient selection to increase the odds of finding beneficial therapies for specific patient cohorts.

## 5. Conclusions

Organoids offer distinct advantages compared to previous in vitro methods of culture for human tissue, including recapitulation of three-dimensional tissue-specific structures, cell–cell and cell–stroma interactions, and genetic and cellular heterogeneity. In the decade following the first description of normal gastrointestinal tissue organoids, these models have been produced in multiple tissue types and cancers. PDOs can be created from fresh tumor tissue samples and produced rapidly compared to other cancer modeling systems. Early data suggest that PDOs can be produced in clinically relevant time frames, recapitulate characteristic biologic features of the parent tumor, and respond to oncologic therapy similarly to the parent tumors. These advantages may provide an avenue for the implementation of drug-screening approaches that allow clinicians to test tumor responsiveness to multiple chemotherapy, radiation, and targeted cancer treatment modalities. The recent development of PDOs for GBM is also important, given the historical limitations of brain tumor culture and xenograft models. While initial studies on PDOs are promising, limitations of cancer organoid model implementation in clinical oncology care include variable success rates in PDO establishment, sparse knowledge on the persistence of tumor-associated immune cells in PDOs, and limited prospective, comparative clinical data. Given the current trajectory of research in this field, PDOs appear poised to contribute to the implementation of precision oncology care for patients.

## Figures and Tables

**Table 1:** Culture conditions used for tumor organoid creation.

Cancer Type	Source of Organoids	Culture Technique	Endpoint of Study	Resemblance to Parent Tumor	References
Bladder cancer	Human bladder cancer cells	Matrigel + culture with hepatocyte medium, charcoal-stripped serum, and ROCK inhibitor	Patient-derived bladder cancer organoids able to recapitulate histologic findings of primary tumors	Histopathologic analysis of tumor organoids showed concordance to parental tumors.	Lee et al. (2018) [59]
Breast cancer	Human breast cancer single cells	Culture with medium including mitogen Neuregulin 1	Protocol for breast cancer organoid creation	Blinded histologic analysis showed concordant lobular and ductal carcinoma and breast cancer biomarkers.	Sachs et al. (2018) [28]
	Human breast cancer tissue	Matrigel or 3D collagen I + culture with medium containing insulin, EGF, hydrocortisone, and cholera toxin	Identified expression of cytokeratin-14 and p63 important for invasion of poorly differentiated carcinomas	Cytokeratin-14+ cells were observed in primary tissue and corresponding organoids.	Cheung et al. (2013) [73]
	Normal and tumor breast cancer cells	Three-dimensional bioprinting	Three-dimensional bioprinting able to create multicellular, architecturally defined, scaffold-free organoid models	Bioprinted organoids retained a similar structural morphology to the parental tumor.	Langer et al. (2019) [74]
Clear-cell renal cell carcinoma	Human clear-cell renal cell carcinoma single cells	Kidney three-dimensional medium + Matrigel and cultured for organoid formation	Patient-derived clear-cell renal cell carcinoma organoids demonstrating a high degree of genetic similarity to primary tissue	PDOs were concordant to primary tissue through VHL sequencing.	Bolck et al. (2019) [75]
Gastrointestinal tract cancers	Mouse- and human-derived adult stem cells, adult epithelial crypts, and colon cancer cells	Matrigel + culture media containing human Wnt-3A	Refined protocol for the culture and long-term culture of colon adenocarcinoma organoids	Similar levels of LGR5 and Axin2 were observed on day 7 of organoid culture compared to fresh adenoma tissue.	Sato et al. (2009) [36] Sato et al. (2011) [48]
	Mouse and human adult stem cells and mouse neonatal gut	Organoids derived from genetically engineered mice using a single air–liquid interface culture approach	Gastrointestinal primary tissue epithelial and mesenchymal organoids reprogrammed to model cancer formation and oncogene activation	Organoids displayed a well-differentiated epithelial layer of surface mucous cells.	Li et al. (2014) [76]
	Human induced pluripotent stem cells	CRISPR/Cas9-mediated genome editing to normal human small-intestinal organoid stem cell cultures	Creation of colorectal cancer organoids through genetic modification of normal intestinal epithelium organoids to study disease	Orthotopically implanted genetically engineered organoids displayed large cysts and well-differentiated carcinomas.	Drost et al. (2015) [49] Matano et al. (2014) [50]
	Human colorectal cancer tissue	Followed Sato et al.’s (2011) protocol	Tested the sensitivity of cetuximab based on molecular characterization of patient-derived colorectal cancer organoids	There was no direct comparison to primary tissue.	Verissimo et al. (2016) [51]
	Human colorectal cancer tissue	Followed Sato et al.’s (2011) protocol	Patient-derived colorectal tumor organoids protocol able to recapitulate somatic copy number and mutations found in colorectal cancer primary tissue	All studies found concordance of genetic diversity in organoids and parent tumor with DNA sequencing.	Van de Wetering et al. (2015) [30] Weeber et al. (2015) [52] Roerink et al. (2018) [46]
	Human esophageal adenocarcinoma cells	BME + culture	Patient-derived esophageal adenocarcinoma organoid protocol created	Organoids displayed normal glandular architecture and wild-type p53 expression consistent with parent gastric tissue.	Li et al. (2018) [55]
	Human gastric cancer cells	Matrigel + culture with media supplemented with ROCK inhibitor Y-27632	Patient-derived gastric cancer organoids having similar immunohistochemical and mutational profiles as primary gastric cancer tissue	PDOs displayed cytokeratin 7, cadherin 17, carcinoembryonic antigen, and periodic acid Schiff reaction similar to parent tissue.	Seidlitz et al. (2017) [77]
	Human gastric cancer tissue	Established based on Barfeld et al.’s (2015) protocol; cultured in media containing Nutlin-3, ROCK inhibitory free medium, TGF-β, and absence of EGF and FGF-10	Patient-derived gastric cancer organoid biobank established to investigate the role of driver gene mutations in gastric cancer	Histologic and genomic analysis of the organoid displayed concordance to parent tissue.	Nanki et al. (2018) [78]
	CDH1 and Trp53 murine gastric cancer tissue	Collagen gel + double-dish air–liquid interface culture system	Murine-derived gastric cancer organoids to study the role of TGF-β receptor 2 loss of function mutation in metastatic transformation of diffuse gastric cancer	Organoids displayed similar copy number variation, allelic imbalances, and rearrangements as primary tissue.	Nadauld et al. (2014) [64]
	Human gastrointestinal cancer cells	Matrigel + culture with human organoid media	Established organoid biobank for patient-derived metastatic and/or relapsed colorectal and gastroesophageal cancer	Colorectal organoids retained diffuse and intestinal growth patterns.	Vlachogiannis et al. (2018) [79]
Liver cancer	Human primary liver cancer cells	BME + culture with media containing 3 nM dexamethasone	Hepatocellular carcinoma, cholangiocarcinoma, and combined hepatocellular carcinoma/cholangiocarcinoma organoid model creation	Organoids formed pseudo-glandular rosettes consistent with parent hepatocellular carcinoma.	Broutier et al. (2017) [25]
Non-small-cell lung cancer	Human non-small-cell lung cancer cells	BME + culture with media containing Nutlin-3a used to select for specific cancer organoids	Demonstrated the ability of lung tumor organoids to recapitulate histopathologic and genetic features of primary tissue	Organoids recapitulated histopathologic features of adenocarcinoma, mucinous adenocarcinoma, and large-cell neuroendocrine parent tumors.	Sachs et al. (2019) [65]
Ovarian cancer	Human induced pluripotent stem cells	Matrigel + cultured according to protocols by Xia et al., May et al., and Takasoto et al.	Conditions to create fallopian tube epithelial organoids described to study high-grade serous ovarian carcinoma	Organoids expressed TUB4A, FOXJ1, and PAX8 consistent with normal fallopian tubes.	Yucer et al. (2017) [47]
	Human ovarian cancer cells	BME + culture in media containing hydrocortisone, forskolin, and heregulin β-1	Protocol established for creation and propagation of ovarian cancer organoids	Expression of markers of secretory and ciliated cells was shared with primary tissue.	Kopper et al. (2019) [27]
Pancreatic cancer	Murine pancreatic ductal cells and human malignant pancreatic tissue	Matrigel + culture with media containing Wnt3a, Noggin, epidermal growth factor, gastrin, fibroblast growth factor 10, gastrin, nicotinamide, and A83-01	Culture conditions refined to passage and transplant pancreatic organoids for molecular and cellular biology analysis	PDOs had elevated CA 19-9 expression consistent with parent tumor tissue.	Boj et al. (2015) [24]
	Human pancreatic ductal cancer tissue	Culture with pancreatic progenitor and tumor organoid medium	Conditions refinedto support tumor organoid growth from patient-derived pancreatic ductal adenocarcinoma organoids	Organoids expressed NKX6.1 and PTF1A, pancreas-specific markers, on gene expression analysis.	Huang et al. (2015) [80]
	Human pancreatic adenocarcinoma cells	Matrigel + culture with media containing Nutlin-3 and BMP-4, absent of EGF and Noggin	Pattern of dependency on Wnt ligands determined for pancreatic cancers using patient-derived organoids	Organoids contained common driver gene alterations, including KRAS, CDKN2A, TP53, and SMAD4, on whole-exome sequencing and comparative genomic hybridization analyses.	Seino et. Al. (2018) [81]
	Human pancreatic cancer cells	Matrigel + culture in human complete feeding medium	Establishment of a patient-derived pancreatic cancer organoid library	Whole-exome sequencing of organoids and PDOs was performed.	Tiriac et al. (2018) [66]
Prostate cancer	Genetically engineered murine prostate epithelial cells and human prostatic epithelium	Matrigel + culture with media containing EGF, Noggin, R-spondin 1, FGF-10, FGF-2, prostaglandin E2, nicotinamide, and p38 inhibitor SB202190	Creation of a culture system for long-term expansion of murine and human prostatic epithelial organoids	Organoids displayed a cystic structure and expressed basal prostate markers, including p63, Ck5, and Ck8.	Karthaus et al. (2014) [60]
	Human induced pluripotent stem cells	Matrigel + culture with growth factors	Prostate cancer organoid protocol description	PDOs maintained typical histologic patterns of prostate adenocarcinomas.	Gao et al. (2014) [26]

Abbreviations: EGF, epidermal growth factor; FGF, fibroblast growth factor; TGF, transforming growth factor; BMP, bone morphogenic protein; BME, basement membrane extract.

**Table 2:** Glioma organoid models.

Cancer Type	Source of Organoids	Culture Technique	Endpoint of Study	Resemblance to Parent Tumor	References
Glioblastoma	Patient-derived glioma stem cells	1. Co-culture of GSCs and iPSCs 2. Supplementing GSCs with normal cerebral organoids 3. Fusion of GSC spheres with normal brain organoids	Three techniques to model GSC invasion in normal brain organoids and creation of GBM organoids	No direct comparison to primary tissue could be made.	Goranci-Buzhala et al. (2020) [83]
	Patient-derived glioblastoma and non-glioblastoma stem cells	Matrigel + culture shaking in NBM complete media	Spatial distribution of GBM replicated in organoids derived from glioblastoma and non-glioblastoma stem cells	Orthotopically implanted PDOs were diffuse and infiltrative, histologically resembling the parent tumor.	Hubert et al. (2016) [57]
	Human embryonic stem cells	Normal cerebral organoids co-cultured with patient-derived tumor cells or oncogene introduction through electroporation	Normal human-derived cerebral organoids a vector for glioblastoma organoid modeling	Engineered PDOs displayed a mesenchymal phenotype consistent with patient-derived GBMs on transcriptomic analysis.	Ogawa et al. (2018) [56]
	Human glioblastoma tissue	Patient tissue parcellated and cultured without an extracellular matrix	Glioblastoma organoid protocol development from primary tissue samples with minimum processing	PDOs had cellular and nuclear atypia, abundant mitotic figures, and pleomorphic nuclei consistent with high-grade parent tumors on histologic analysis.	Jacob et al. (2020) [62]
	Glioblastoma spheroids infiltrating cerebral organoids	Co-culture of mouse early (6-day-old) cerebral organoids with glioblastoma spheroids created from glioblastoma stem cell culture	Demonstration of hybrid glioblastoma organoid modeling	Co-cultured organoids displayed core infiltration and expression of GBM stem cell markers NESTIN and SOX2.	Da Silva et al. (2018) [82]
	Patient-derived glioma stem cells	Co-culture of cerebral organoids with transduced GSCs on NBM	Single-cell RNA sequencing comparison of four patient-derived glioblastoma models	Organoids displayed microscopic invasion and single-cell heterogeneity consistent with parent tumors.	Pine et al. (2020) [85]
Central nervous system primitive neuroectodermal-like and glioblastoma-like tumor	Human embryonic stem cells	Neoplastic cerebral organoids (Neo-COR); combination of plasmids introduced into normal cerebral organoids through electroporation before te organoids embedded in Matrigel	Demonstration of brain tumorigenesis through introduction of oncogenic mutations in normal cerebral organoids through transposon and CRISPR/Cas9 mutagenesis	GBM-like Neo-COR displayed upregulation of GBM genes on transcriptomic analysis.	Bian et al. (2018) [68]

Abbreviations: GBM, glioblastoma; GSCs, glioma stem-like cells; NBM, neurobasal medium.

## Data Availability

Not applicable.
